# Neuroprotective Effect of Pseudoginsenoside-F11 on a Rat Model of Parkinson's Disease Induced by 6-Hydroxydopamine

**DOI:** 10.1155/2013/152798

**Published:** 2013-12-10

**Authors:** Jian Yu Wang, Jing Yu Yang, Fang Wang, Shi Yuan Fu, Yue Hou, Bo Jiang, Jie Ma, Cui Song, Chun Fu Wu

**Affiliations:** Department of Pharmacology, Shenyang Pharmaceutical University, Shenyang 110016, China

## Abstract

Pseudoginsenoside-F11 (PF11), a component of *Panax quinquefolism* (American ginseng), plays a lot of beneficial effects on disorders of central nervous system. In this paper, the neuroprotective effect of PF11 on Parkinson's disease (PD) and the possible mechanism were investigated in a rat PD model. PF11 was orally administered at 3, 6, and 12 mg/kg once daily for a period of 2 weeks before and 1 week after the unilateral lesion of left medial forebrain bundle (MFB) induced by 6-hydroxydopamine (6-OHDA). The results showed that PF11 markedly improved the locomotor, motor balance, coordination, and apomorphine-induced rotations in 6-OHDA-lesioned rats. The expression of tyrosine hydroxylase (TH) in substantia nigra (SN) and the content of extracellular dopamine (DA) in striatum were also significantly increased after PF11 treatment. Moreover, significant reduction in the levels of striatal extracellular hydroxyl radical (^**∙**^OH), detected as 2,3- and 2,5-dihydroxy benzoic acid (2,3- and 2,5-DHBA), and increase in the level of striatal extracellular ascorbic acid (AA) were observed in the PF11-treated groups compared with 6-OHDA-lesioned rats. Taken together, we propose that PF11 has potent anti-Parkinson property possibly through inhibiting free radical formation and stimulating endogenous antioxidant release.

## 1. Introduction

Parkinson's disease (PD), the second most common progressive neurodegenerative disorder, is characterized by degeneration of dopaminergic neurons in the nigrostriatal pathway and subsequent dopamine (DA) depletion in the striatum [1]. Although various mechanisms of neuronal degeneration in PD have been proposed, the exact pathogenesis of PD is not well understood [2, 3]. Increasing studies have demonstrated that one of etiologies about PD is the imbalance between free radical formation and the maintenance of the dopaminergic neuronal integrity through the endogenous antioxidant defense system [4–6]. Ascorbic acid (AA), one of the important antioxidants in the endogenous antioxidant defense system, is thought to play a major role in protecting brain against oxidative damage. It has been reported that the level of plasma AA in PD patient is significantly lower than that in healthy control subject [7]. The previous study in our lab has also proved that release of endogenous AA in the striatum plays an important role in preventing oxidative stress by trapping 2,3- and 2,5-dihydroxy benzoiclacid (2,3- and 2,5-DHBA) that reflected the hydroxy radical (^*∙*^OH) level [8]. It is suggested that AA as an antioxidant may be benefit to PD and be worth further study.

Many experiments have demonstrated that ginseng extracts exert anti-Parkinson effect [9–12]. The previous studies have shown that Pseudoginsenoside-F11 (PF11), an ocotillol-type saponin isolated from leaves of Panax pseudoginseng subsp. *Himalaicus *Hara (Himalayan Panax), but not in *Panax ginseng*, plays an important role in treating many disorders of the central nervous system, such as antioxidation [13], antagonistic actions on morphine and methamphetamine [14–17], and antiamnesic effect [13, 18]. PF11 shows the similar effect to ginsenosides isolated from *Panax ginseng*. Moreover, PF11 is proved to antagonize the decrease of DA in the brain of methamphetamine-treated mice in our previous studies [16]. Considering the improvement of PF11 on DA levels and its antioxidation, it is well worthy to investigate whether PF11, like ginseng saponins, has an antagonistic effect on PD and whether the mechanism is associated with its antioxidative activity.

In the present study, the antagonistic effect of PF11 on 6-OHDA-induced rats of PD model was investigated by using four kinds of behavioral tasks. The level of extracellular DA in striatum and the expression of tyrosine hydroxylase (TH) in substantia nigra (SN), a rate limiting enzyme in the biosynthesis of DA, were also investigated. Furthermore, the protective mechanism of PF11 on PD was also explored by measuring the changes of AA and 2,3- and 2,5-DHBA release in striatum.

## 2. Materials and Methods

### 2.1. Animals

Male Sprague-Dawley rats weighting 200–250 g were supplied by the Experimental Animal Centre of Shenyang Pharmaceutical University and kept with a 12 hL : 12 hD light/dark cycle (light on at 06:30 am). Rats were housed in cages with food and water freely available. All experiments and procedures were carried out according to the Regulations of Experimental Animal Administration issued by State Committee of Science and Technology of China.

### 2.2. Chemicals

PF11 was isolated from the aerial parts of *P. quinquefolism* by the Department of Chemistry for Natural Products of Shenyang Pharmaceutical University. The purity was more than 98% as determined by HPLC. PF11 was dissolved in distilled water before administration. 6-OHDA, apomorphine, salicyliclacid (SA), and its hydroxylated derivatives, 2,3-DHBA and 2,5-DHBA, were purchased from Sigma Chemicals (St. Louis, MO, USA). 6-OHDA was dissolved in saline containing 0.1% AA and prepared freshly in dark to avoid autooxidation. Apomorphine was dissolved in saline. Madopar was purchased from Shanghai Roche Pharmaceuticals Ltd. Ringer's solution (NaCl 147 mM, KCl 4 mM, and CaCl_2_ 2.3 mM) contains 2 mM salicylate as hydroxyl radical trapping reagent [8]. All other chemicals were commercially available and of reagent grade.

### 2.3. Animal Grouping and PF11 Treatment

The animals were divided into 6 groups (6 rats in each group): sham group, 6-OHDA-lesioned group, PF11-treated groups (3, 6, and 12 mg/kg), and Madopar-treated group (50 mg/kg). PF11 and Madopar were orally administered once daily for 3 weeks.

### 2.4. 6-OHDA Lesion

Two weeks after PF11 and Madopar treatment, the left medial forebrain bundle (MFB) was lesioned by 6-OHDA. The rats were anesthetized with chloral hydrate (350 mg/kg, i.p.) and fixed in a stereotaxic instrument. Lesions were made by injecting 6-OHDA (20 *μ*g in saline containing 0.1% AA) into the left MFB at the coordinate: *A*, −2.5; *L*, −2.0; *V*, −8.5 mm, from bregma using a 5 *μ*L Hamilton syringe at a rate of 1 *μ*L/min [19]. The sham animals were injected vehicle only (0.1% AA-saline) at the same coordinate. After injection, the syringe was left in place for an additional 5 min before being slowly retracted. 6-OHDA and sham rats were housed one week for further experiments.

### 2.5. Behavioral Studies

One week after injection of 6-OHDA, animals were subjected to behavior testing, which were, respectively, spontaneous locomotor activity test, rotarod test, narrow beam test, and apomorphine-induced circling test.

#### 2.5.1. Spontaneous Locomotor Activity

The spontaneous locomotor activity of rat was analyzed with a video-tracking system EthoVision (Noldus, Wageningen, NL) [20]. Two animals were placed into neighboring “home” cages under a video camera, respectively. Using the video-tracking system EthoVision, motor behavior was recorded during the monitoring for 5 min. The “total distance” (total distance traveled by the animal during the 5 min observation time, in cm) and the “mean velocity” (mean of the velocities of all 0.2 s periods during the 5 min observation time, in cm/s) were measured.

#### 2.5.2. Rotarod

Rotarod was used for the study of muscular coordination. It consisted of a rotating rod, 75 mm in diameter, which was divided into four parts by compartmentalization to permit the testing of four rats at a time. The time for each rat to remain on the rotating rod was recorded. The speed was set at 10 cycles per min and cut-off time was 180 s. The animals of all groups were trained on rotarod prior to the start of the experiment until they could stay on it at least for the cut-off time. After 1 week of lesioning, the rats of each group were tested on rotarod [21].

#### 2.5.3. Narrow Beam Test

After 1 week of lesioning, the rats were tested for the balance and motor coordination on a narrow beam. The narrow beam had a smooth wooden narrow beam of 105 cm in length, 4 cm in width, and thickness of 3 cm. The beam was elevated from the ground by 1 m with additional supports. It had a goal box at the end of the narrow beam. There was food in the goal box for the reward of the animals. The rats were trained on the narrow beam for 10 trials per day with 1 min interval. The animals were allowed to explore the narrow beam for 10 trials with 1 min interval on 1 day before the experimental day and during this exploration rats were motivated and rewarded with food pellets in the goal box. The time to cross the beam was calculated [21].

#### 2.5.4. Apomorphine-Induced Circling Behavior

The behavior was assessed by monitoring body rotations induced by intraperitoneal injection (i.p.) of apomorphine (0.5 mg/kg). The number of contralateral rotations was recorded for 30 min [22].

### 2.6. Neurochemical Studies

#### 2.6.1. Brain Microdialysis

After behavioral tests, the rats were anesthetized with chloral hydrate (350 mg/kg, intraperitoneal injection) and the dialysis probes (310 *μ*m o.d., 200 *μ*m i.d., AN69, Hospal, Dasco, Bologna, Italy) were implanted through the striatum according to the following coordinates: *A* +1.0 mm from bregma, *V* −5.6 mm from occipital bone. The procedure used to prepare and implant the dialysis probe was the same as previously described [23]. Twenty-four hours after implantation, the rats were perfused with ringer's solution containing 2 mM salicylate as ^*∙*^OH trapping reagent by means of a microinjection pump with a constant rate of 2 *μ*L/min. The first 1 h samples for equilibrium were discarded and then the dialysate was collected for 20 min. Twenty microliters of each collected sample was used for analysis of the levels of AA, while twenty microliters of each collected sample was used for analysis of DHBA levels [8]. Another group of animals were used to collect the dialysate for DA determination. When the experiment was finished, rat was sacrificed for checking the position of the dialysis fiber. The datum was discarded if the fiber was positioned incorrectly [24].

#### 2.6.2. Assay for DA

The level of extracellular DA was determined by high performance liquid chromatography with electrochemical detection (HPLC-ECD) in accordance with a previously described method [25]. This system used a reverse phase column (C-18, 5 *μ*m, Agilent) with a mobile phase composed of 85 mM citrazinic acid, 100 mM sodium acetate anhydrous, 0.2 mM Na_2_EDTA, and 1.2 mM sodium 1-octanesulfonate adjusted to pH 3.7 [25]. The mobile phase was pumped with LC-10A pump (Shimadzu, Japan) at a flow rate of 1.0 mL/min. The electrochemical detector (L-ECD-6A, Shimadzu, Japan) was set at +0.70 V. All the mobile phases were filtered through a 0.22 *μ*m filter. The column temperature was maintained at 37°C.

#### 2.6.3. Assay for AA

The content of extracellular AA was measured using method that has been described by Liu et al. [26]. HPLC-ECD and a reverse phase column (C-18, 5 *μ*m, Agilent) were used with the mobile phase composed of 155.6 mM NaCl, 0.54 mM EDTA-Na_2_, and 1.5 mM tetrabutylammonium bromide as an ion pairing reagent. The flow rate was 1.0 mL/min. The detector was set at +0.60 V.

#### 2.6.4. Assay for DHBA

The samples (20 *μ*L) were injected into HPLC system to detect the contents of 2,3, and 2,5-DHBA to evaluate the ^*∙*^OH level. The mobile phase consisted of 0.03 M citrate, 0.03 M acetate, and 10% methanol [8]. The equipment conditions were the same as that used for detecting AA described above.

### 2.7. Immunohistochemical Study

Immunohistochemistry was performed as previously described [27] with minor modifications. The rats were anesthetized with chloral hydrate (350 mg/kg, i.p.) and perfused transcardially through ascending aorta with 0.1 M phosphate buffer saline (PBS) at pH 7.4 followed by 4% paraformaldehyde in 0.1 M phosphate buffer. Brain was removed immediately and fixed in the same fixative for an additional 24 h at 4°C; furthermore the tissues were preserved in 10%, 20%, and 30% sucrose solution (in PBS) until they sank. The fixed tissues were embedded in OCT compound (polyvinyl glycol, polyvinyl alcohol, and water) and frozen at −20°C. Coronal sections of 20 *μ*m thicknesses were cut on the cryostat (Leica, Germany). The sections were rinsed three times for 5 min in PBS, and endogenous peroxidase activity was blocked with 3% hydrogen peroxide in methanol and incubated for 20 min at room temperature. Slices were permeabilized and blocked with PBS containing 1% Triton X-100 and 4% normal goat serum, for 1 h at 37°C. Thereafter, the sections were incubated in primary antibody (anti-TH mouse, 1 : 1000, Sigma) for 24 h at 4°C, rinsed three times for 5 min in PBS, and subsequently incubated with avidin-biotin-horseradish peroxidase conjugate (ABC Staining System, Santa Cruz Biotechnology) for 1 h. After washing, the slides were incubated with biotinylated rabbit anti-mouse secondary antibody. The color was developed using DAB as a chromogen. Finally, sections were dehydrated in graded alcohol solutions, cleared in xylene, and coverslipped to be viewed under a microscope and photo micrographs were taken using a light microscope (Olympus BX 51, Japan). Three sections per animal from the substantia nigra were selected. The intensity of the TH immunoreactivity at ×400 magnification was measured by semiquantitative densitometric analysis using an image-analysis program (Image-Pro Plus Version 6.0, Media Cybernetics, USA).

### 2.8. Statistical Analysis

Statistical analysis was carried out by SPSS 13.0 software for windows (SPSS Inc., Chicago, IL, USA). All values were expressed as mean ± S.E.M. Data were analyzed via one-way analysis of variance (ANOVA) followed by Dunnett's *t*-test. Statistical significance was accepted at *P* < 0.05.

## 3. Results

### 3.1. Neurobehavioral Studies

#### 3.1.1. The Effect of PF11 on the Spontaneous Locomotor Activity

**Figure 1:**
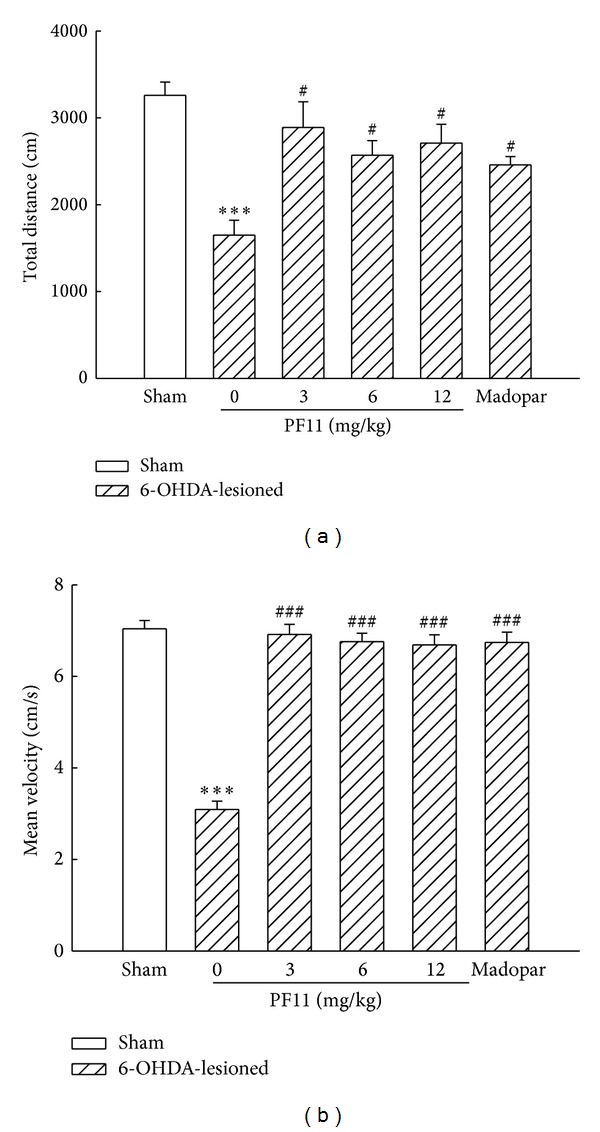
Effect of PF11 on spontaneous activity in 6-OHDA-lesioned rats. Results were means ± SEM of the data obtained in six rats per group. ***P < 0.001 compared to sham group; ^#^ *P* < 0.05, ^###^ *P* < 0.001 compared to 6-OHDA-lesioned group.

As shown in Figure 1(a), one week after the 6-OHDA lesion, the total distance was typically decreased in the 6-OHDA-lesioned rats compared with the sham group (*P* < 0.001). However, the decrease of total distance was significantly blocked by PF11 (3, 6, and 12 mg/kg) (*P* < 0.05).  Figure 1(b) showed that a slower mean velocity in the 6-OHDA lesioned rats was observed (*P* < 0.001). There was significant increase in the mean velocity in PF11-treated groups compared with the 6-OHDA-lesioned group (*P* < 0.001). The mean velocity was improved and almost returned to the level of sham group after all of the doses of PF11 administration. These data indicated that PF11 significantly improved the impaired spontaneous locomotor activity in PD rats. Madopar-treated group (50 mg/kg) also showed improvement in total distance and mean velocity (*P* < 0.05 and *P* < 0.001).

#### 3.1.2. The Effect of PF11 on the Muscular Coordination

**Figure 2:**
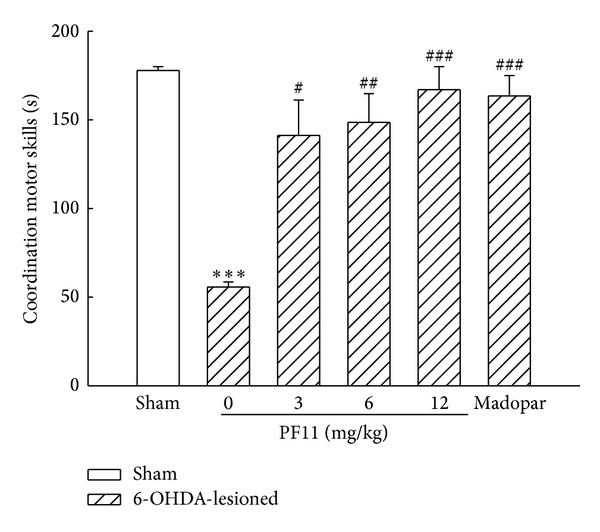
Effect of PF11 on the muscular coordination in 6-OHDA-lesioned rats. Results were means ± SEM of the data obtained in six rats per group. ***P < 0.001 compared to sham group; ^#^ *P* < 0.05, ^##^ *P* < 0.01, and ^###^ *P* < 0.001 compared to 6-OHDA-lesioned group.

The rotarod test was used for the evaluation of muscular coordination in the present study. A significant depletion (*P* < 0.001) in muscular coordination in 6-OHDA-lesioned group was observed as compared with sham group (Figure 2). PF11 (3, 6, and 12 mg/kg) was found to be effective in recovery of muscular coordination in a dose-dependent manner (*P* < 0.05, *P* < 0.01, and *P* < 0.001). The increase of muscular coordination was also observed in Madopar-treated group (50 mg/kg) (*P* < 0.001).

#### 3.1.3. The Effect of PF11 on the Motor Coordination

**Figure 3:**
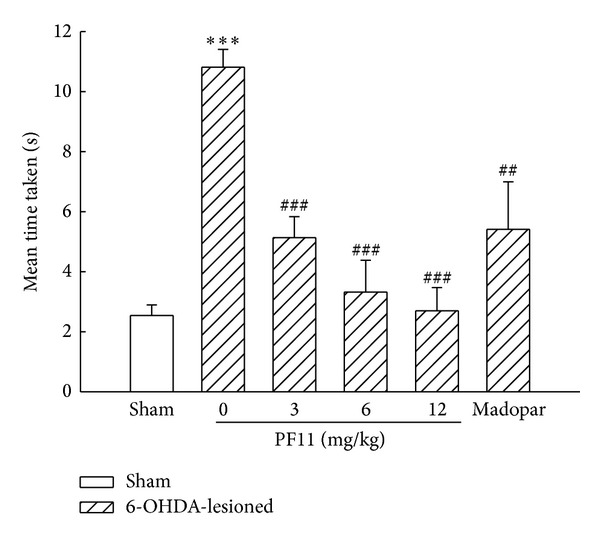
Effect of PF11 on the motor coordination in 6-OHDA-lesioned rats. Results were means ± SEM of the data obtained in six rats per group. ***P < 0.001 compared to sham group; ^##^ *P* < 0.01, ^###^ *P* < 0.001 compared to 6-OHDA-lesioned group.

In the present study, the rats were tested for the balance and motor coordination on the narrow beam. The time taken to cross the beam was significantly increased in 6-OHDA-lesioned rats when compared with the sham group (*P* < 0.001). PF11 (3, 6, and 12 mg/kg) markedly decreased the time taken to cross the beam in a dose-dependent manner (*P* < 0.001), indicating that PF11 showed significant improvement in balance ability (Figure 3). The increase of time taken to cross the beam was shown in Madopar-treated group (50 mg/kg) (*P* < 0.01).

#### 3.1.4. The Effect of PF11 on the Rotation Induced by Apomorphine

**Figure 4:**
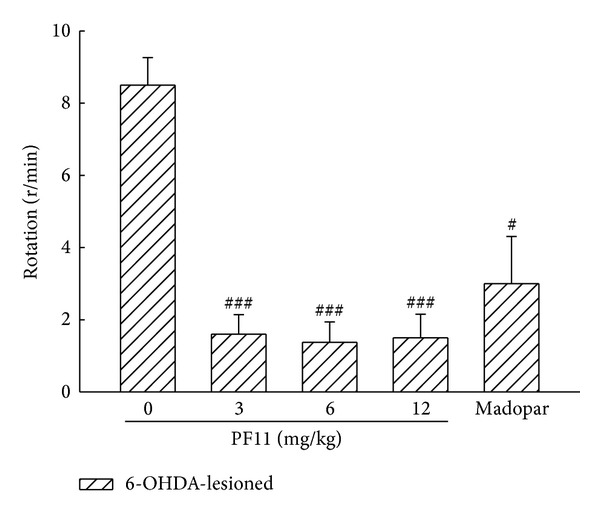
Effect of PF11 on rotation behavior in 6-OHDA-lesioned rats. Results were means ± SEM of the data obtained in six rats per group. ^#^ *P* < 0.05, ^###^ *P* < 0.001 compared to 6-OHDA-lesioned group.

Rotation induced by apomorphine was usually used to behaviorally assay the extent of neuronal loss following lesion by 6-OHDA. No rotation was observed in all rats in sham group. Significant increases in the number of apomorphine-induced rotations were observed in the 6-OHDA-lesioned rats compared to the sham group (*P* < 0.001). As shown in Figure 4, rats receiving PF11 (3, 6, and 12 mg/kg) exhibited significant attenuation (*P* < 0.001) in circling behavior, indicating that PF11 significantly reversed this abnormal motor behavior of 6-OHDA. The improvement of circling behavior was also observed in Madopar-treated group (50 mg/kg) (*P* < 0.05).

### 3.2. Neurochemical Studies

#### 3.2.1. The Effect of PF11 on the Level of Extracellular DA in Striatum

**Figure 5:**
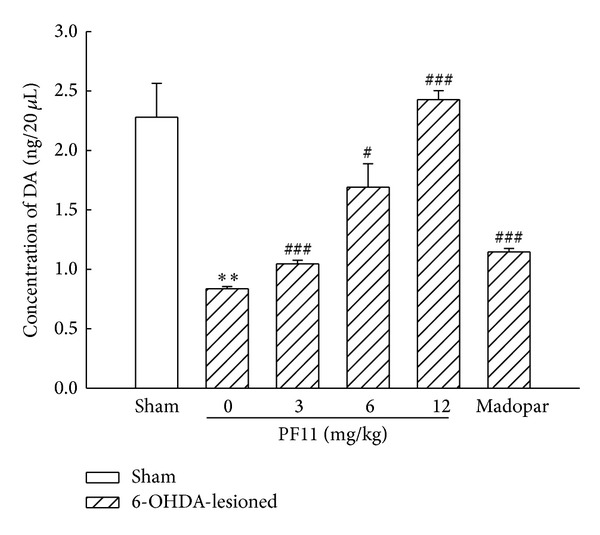
Effect of PF11 on the release of striatal DA in 6-OHDA-lesioned rats. Results were means ± SEM of the data obtained in six rats per group. **P < 0.01 compared to sham group; ^#^ *P* < 0.05, ^###^ *P* < 0.001 compared to 6-OHDA-lesioned group.

The effect of PF11 on the level of extracellular DA in the striatum was analyzed by using microanalysis coupled with HPLC-ECD. The level of striatal extracellular DA was lower in the 6-OHDA-lesioned group than that in the sham group (*P* < 0.01). Administration of PF11 (3, 6, and 12 mg/kg) significantly increased the level of striatal extracellular DA in a dose-dependent manner (*P* < 0.05 and *P* < 0.001) (Figure 5) and PF11 (12 mg/kg) significantly recovered the level of striatal extracellular DA to the normal level. The improvement of striatal DA was also shown in Madopar-treated group when compared with 6-OHDA-lesioned group (*P* < 0.001).

#### 3.2.2. The Effect of PF11 on the Level of Extracellular AA in Striatum

**Figure 6:**
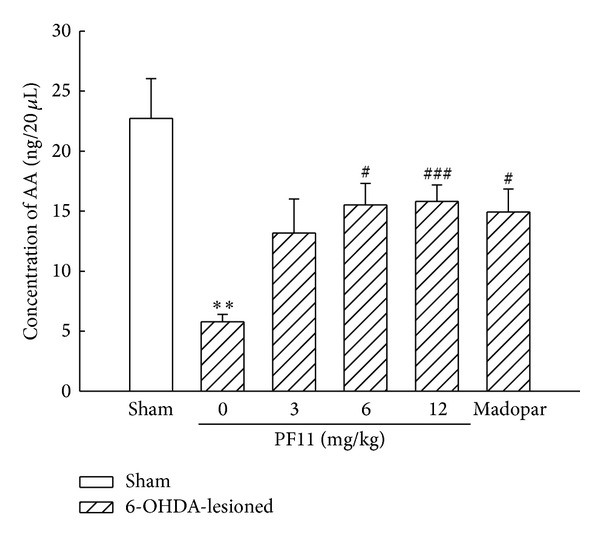
Effect of PF11 on the release of striatal AA in 6-OHDA-lesioned rats. Results were means ± SEM of the data obtained in six rats per group. **P < 0.01, compared to sham group; ^#^ *P* < 0.05, ^###^ *P* < 0.001 compared to 6-OHDA-lesioned group.

AA was an important endogenous antioxidant which was involved in cellular protection against damage caused by oxidative stress. As presented in Figure 6, a markedly lower level of striatal extracellular AA was exhibited in 6-OHDA-lesioned group compared to the sham group (*P* < 0.01). The PF11-treated rats (6, 12 mg/kg) attenuated the 6-OHDA-induced striatal extracellular AA depletion (*P* < 0.05, *P* < 0.001), indicating that PF11 could enhance the antioxidant ability in striatum of PD rats. Madopar-treated group (50 mg/kg) also showed improvement in the level of striatal extracellular AA (*P* < 0.05).

#### 3.2.3. The Effect of PF11 on the Level of Extracellular DHBA in Striatum

**Figure 7:**
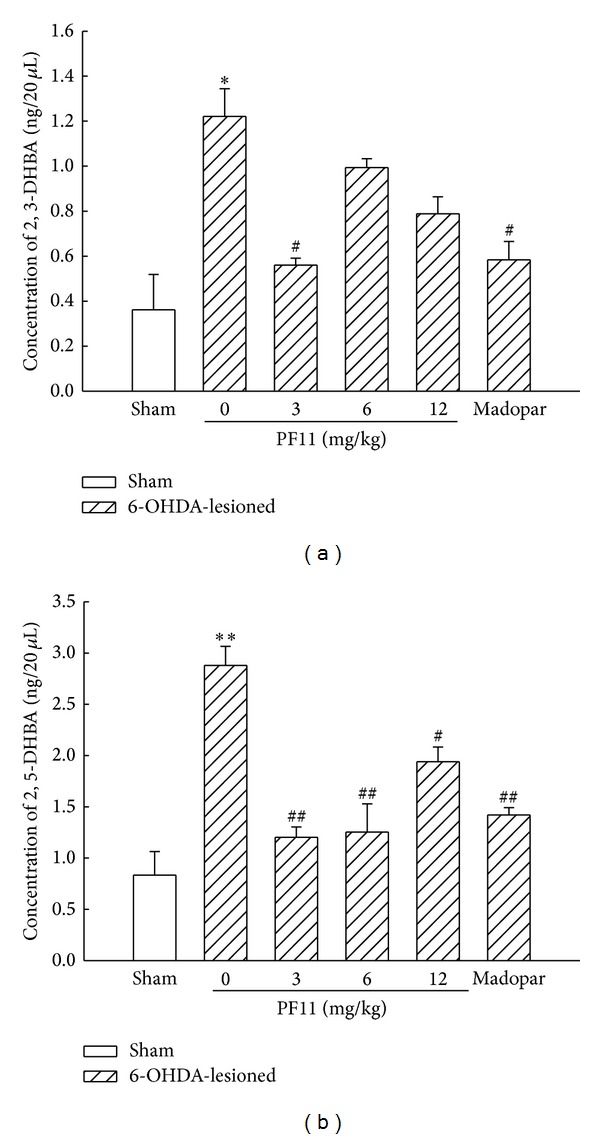
Effect of PF11 on the levels of striatal 2,3- and 2,5-DHBA in 6-OHDA-lesioned rats. Results were means ± SEM of the data obtained in six rats per group. *P < 0.05, **P < 0.01 compared to sham group; ^#^ *P* < 0.05, ^##^ *P* < 0.01 compared to 6-OHDA-lesioned group.

In order to detect the effect of PF11 on the level of extracellular DHBA in striatum, the levels of striatal extracellular 2,3-DHBA and 2,5-DHBA, major products of ^*∙*^OH interacted with salicylic acid, were detected. As shown in Figures 7(a) and 7(b), the contents of extracellular 2,3-DHBA and 2,5-DHBA in striatum were significantly increased in 6-OHDA-lesioned group compared with sham group (*P* < 0.05 and *P* < 0.01). Following three weeks of treatment with PF11, the increase of striatal extracellular 2,3-DHBA was significantly inhibited by PF11 (3 mg/kg) (*P* < 0.05) (Figure 7(a)) and the increase of striatal extracellular of 2,5-DHBA was also significantly decreased by PF11 (3, 6, 12 mg/kg) (*P* < 0.01, *P* < 0.01, and *P* < 0.05). The contents of 2,3-DHBA and 2,5-DHBA were decreased in Madopar-treated group (50 mg/kg) when compared to 6-OHDA-lesioned group (*P* < 0.05, *P* < 0.01).

### 3.3. The Effect of PF11 on the Expression of TH in SN

**Figure 8:**
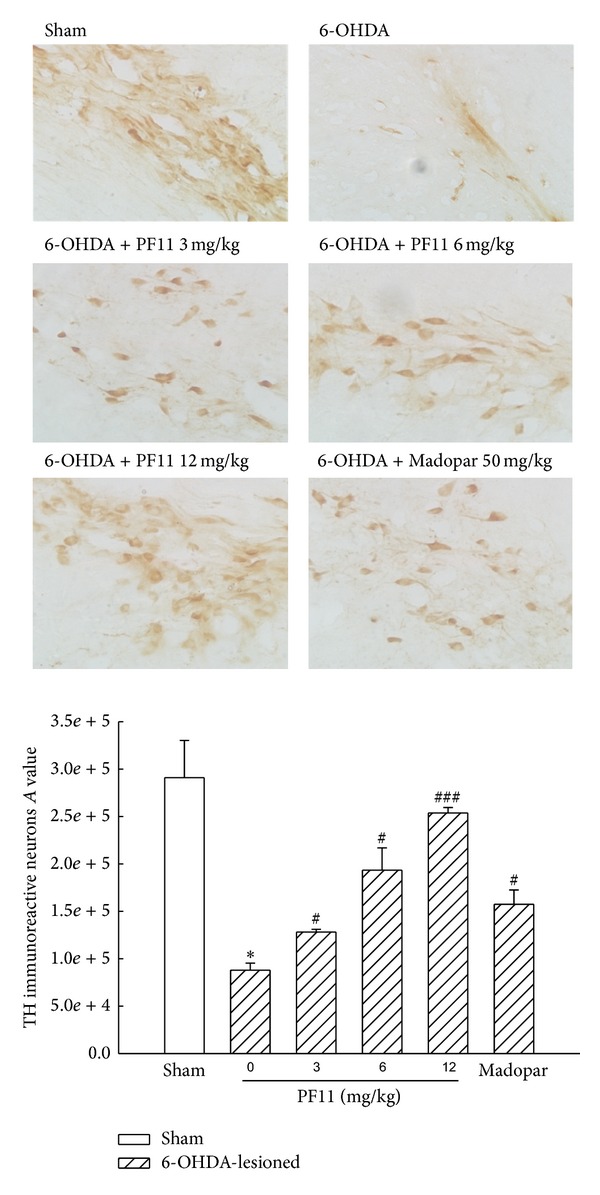
Effect of PF11 on the TH immunoreactive neurons in the left SN of 6-OHDA-lesioned rats. Photomicrographs showed the morphology of the SN neurons in the sham rats, 6-OHDA-lesioned rats, PF11-treated rats (3, 6, and 12 mg/kg), and Madopar-treated rats. The magnification was 400X. Results were means ± SEM of the data obtained in six rats per group. *P < 0.05 compared to sham group; ^#^ *P* < 0.05, ^###^ *P* < 0.001 compared to 6-OHDA-lesioned group.

Just as shown in Figure 8, immunohistochemical analysis showed that a marked depletion in the expression of TH in the left SN in 6-OHDA-lesioned group compared to the sham group (*P* < 0.05) and a pronounced restoration were observed in the PF11-treated groups (3, 6, and 12 mg/kg) in a dose-dependent manner (*P* < 0.05, *P* < 0.001). Madopar (50 mg/kg) decreased the loss of TH immunoreactivity compared with 6-OHDA-lesioned group (*P* < 0.05). The result showed that PF11 increased the number of dopaminergic neurons in PD rats.

## 4. Discussion

The results of this study clearly showed that the protective effect of PF11 on a rat model of PD is induced by 6-OHDA. It was found that treatment with PF11 for three weeks improved performances in four kinds of behavior test, increased the level of extracellular DA in striatum, and inhibited the loss of DA neuron in SN. Because 6-OHDA can be easily carried inside the dopaminergic neurons by the dopamine transporter (DAT), it is thought to be one of the most common neurotoxins used in degeneration models of the nigrostriatal dopaminergic system, in vivo and in vitro [28–30]. In this study, we used unilateral injection of 6-OHDA into MFB to establish a rat model of PD which mimicked behavioral, biochemical, and histopathological abnormalities observed in patients with PD [31].

In the present study, treatment with PF11 at all 3 doses for 3 weeks significantly improved the abnormal behaviors in PD rats induced by 6-OHDA, including the locomotion, muscle and motor coordination, and contralateral rotation induced by apomorphine. Some of them even almost recovered to the levels of sham rats. Our results suggested that PF11 had potent anti-Parkinson property. The doses of PF11 were selected in the present study according to our previous studies which showed that PF11 had protective effect on memory impairment [18]. Our previous studies have proved that PF11 can antagonize the decreases in striatal DA levels in mice induced by methamphetamine [16]. It is reported that methamphetamine and its derivatives may be one of the main causes of PD observed in abusers of these substances, because of their neurotoxicity [16]. Acute administration of a high dose or multiple administration of methamphetamine in a short period can induce tardive dyskinetic movements and depletion of DA content from dopaminergic neurons in man [16]. It is suggested that PF11 possibly has anti-Parkinson property through improving the function of dopaminergic nerve. In the present study, PF11 was found to increase significantly striatal extracellular DA level and the expression of TH in SN in PD rats. Tyrosine hydroxylase is usually thought to be a rate-limiting enzyme in the synthesis of DA, and its expression is the marker for the DA neuron survival. Combined with previous studies and our present study, it is suggested that PF11 may have the neuroprotective action on PD by improving degeneration of dopaminergic neurons in the SN and DA depletion in the striatum.

In order to explore the possible mechanism about the effect of PF11 on PD, we detected the contents of striatal extracellular 2,3- and 2,5-DHBA and AA. It has been demonstrated that dopaminergic neurons in PD are especially vulnerable to oxygen free radicals [32]. Oxygen free radicals, especially ^*∙*^OH, may be a kind of important factor in the onset and/or progression of PD and may finally contribute to neuronal cell death [33]. 6-OHDA-induced dopaminergic neuron degeneration involves the processing of hydrogen peroxidase and ^*∙*^OH [34]. Furthermore, it has been reported that 6-OHDA lesion decreases striatal glutathione (GSH) and superoxide dismutase (SOD) enzyme activity [35] and increases level of malondialdehyde [36]. Interestingly, endogenous 6-OHDA has been found to be accumulated in patients with PD [37]. Taken together, in neurodegenerative processes, 6-OHDA causes oxidative stress, induced by ^*∙*^OH formation, which can damage mitochondria and other cellular components and ultimately causes dopaminergic neuron death [38]. Evidence for the depletion of antioxidants and antioxidant enzymes accounts for the oxidative stress associated with 6-OHDA toxicity in PD [22]. Therefore, it is important to protect DA neurons degeneration through increasing the function of antioxidant defense systems. Our previous study showed that PF11 restored the activities of SOD and glutathione peroxidase (GSH-Px) and decreased the production of malondialdehyde (MDA) in the cortex of APP/PS1 mice [13]. It suggests that enhancing the function of antioxidant system may be one of the mechanisms of the central neuroprotective effects of PF11. It is well known that AA, a classical endogenous antioxidant, plays a major role in protecting brain against oxidative damage in the central nervous system [39, 40]. AA can directly scavenge oxygen- or nitrogen-based radical species generated during normal cellular metabolism. At the millimolar concentrations present in neurons in vivo, AA can effectively scavenge superoxide, a major diffusible byproduct of rapid neuronal mitochondrial metabolism [41], and also compensate for loss of any other single component of the antioxidant network [42]. Consequently, it is especially important to study the change of AA level in PD rats. Our study firstly found that the level of striatal AA was significantly decreased in PD rats, and PF11 markedly increased the content of striatal AA. In recent years, AA has been considered as a neuromodulator or neuroprotectant and plays a significant role in normal neuronal physiology [39]. For instance, AA involves in the synthesis of DA and also induces synaptic maturation of the neurons [41]. These ascorbate effects are not mimicked by other antioxidants, such as GSH and vitamin E [41]. Our study indicated that the effect of PF11 on the increase of DA release and the expression of TH might be due to the increase of AA which could protect the DA neurons.

AA depletion might cause inability to scavenge free radicals resulting in lipid peroxidation, leading to more oxidant load and further oxidative damage [41]. To observe the change of ^*∙*^OH after PF11 treatment in PD model, we detected the levels of 2,3- and 2,5-DHBA, which were thought to be the sensitive assays for ^*∙*^OH formation both in vitro and in vivo [8] by using microanalysis coupled with HPLC-ECD. We found that the levels of striatal extracellular 2,3- and 2,5-DHBA were much higher in PD rats than those in the sham rats. PF11 treatment significantly reduced the increase of 2,3- and 2,5-DHBA in the striatum. The above results about ^*∙*^OH and AA suggested that PF11 protected dopaminergic nerve damage induced by 6-OHDA possibly via direct free radical scavenging or indirect free radical scavenging by enhancing the function of AA.

It should be noticed that several pathogeneses are involved in PD [2]. For example, glutamate excitation and neuroinflammation induced by 6-OHDA are the important factors involved in the pathogenesis of PD [3]. Our previous experiments have shown that PF11 improves the change of glutamic acid in the medial prefrontal cortex of mice induced by morphine [17] and has antiamnesic effect in mouse model of Alzheimer's disease through antioxidation and antiapoptosis [13]. Moreover, it is reported that ginsenoside Rd inhibits the neuroinflammation of dopaminergic neurons evoked by lipopolysaccharide exposure [43]. Whether the neuroprotective effect of PF11 on PD is related to glutamate and neuroinflammation, needs to be studied in detail in the future.

In conclusion, our results showed that PF11 reduced the loss of nigral dopaminergic neurons, promoted the release of striatal DA, and subsequently improved the abnormal behaviors in PD rat model. Furthermore, PF11 treatment significantly reduced the DHBA level and increased the content of striatal AA in PD rats, indicating that PF11 had neuroprotective effect on 6-OHDA-induced PD through scavenging directly free radical and enhancing the antioxidant ability. PF11 may serve as a promising clinical agent in the treatment of PD.
